# Regulatory effects of *Lactobacillus plantarum*-GMNL6 on human skin health by improving skin microbiome

**DOI:** 10.7150/ijms.51545

**Published:** 2021-01-01

**Authors:** Wan-Hua Tsai, Chia-Hsuan Chou, Ying-Ju Chiang, Ching-Gong Lin, Che-Hsin Lee

**Affiliations:** 1 Research and Development Department, GenMont Biotech Incorporation, Tainan, Taiwan; 2 Bachelor Program in Cosmeceutical and Biotech industry, Department of Cosmetic Science, Chia Nan University of Pharmacy & Science; 3 Department of Biological Sciences, National Sun Yat-sen University, Kaohsiung, Taiwan; 4 Department of Medical Research, China Medical University Hospital, China Medical University, Taichung, Taiwan; 5 Department of Medical Laboratory Science and Biotechnology, Kaohsiung Medical University, Kaohsiung, Taiwan

**Keywords:** *Lactobacillus plantarum*-GMNL6, probiotics, skin care, care products

## Abstract

Bacteria response to their environment by producing some compounds which are used in cosmetic and pharmaceutical applications. Some probiotics can regulate immune response and modulate the symptoms of several diseases. Bacteria affect skin response to skin care products. Bacteria are thought to play an important role in acne incidence, skin moisture, and nutrient metabolism, but only a few studies have focused on the extracts of *Lactobacillus plantarum* in skin care. In this study, we identified that *L. plantarum-*GMNL6 enhanced collagen synthesis and the gene expression of serine palmitoyltransferase small subunit A. Meanwhile, *L. plantarum-*GMNL6 reduced the melanin synthesis, the biofilm of *Staphylococcus aureus*, and the proliferation of *Cutibacterium acnes.* Information from clinical observation during the ointment for external face use in people displayed that the syndromes of skin moisture, skin color, spots, wrinkles, UV spots, and porphyrins were improved. The diversification of human skin microbiomes was affected by smearing the face of volunteers with* L. plantarum*-GMNL6. Understanding the potential mechanisms of the action of *L. plantarum*-GMNL6 in dermatologic conditions promotes the development of care products.

## Introduction

Some products of bacteria with anti-oxidative activity have been show to play an important role in skin care. Bacteria can produce some compounds in response to environmental stress. These compounds of bacteria are generally used in medicine, cosmetics, even in sports 1. Some probiotics have immunomodulatory activities and modulate the symptoms of several diseases 2. Previously, the extracts of *Rhodobacter sphaeroides* inhibited inducible nitric-oxide synthase expression in activated macrophages, and reduced inflammation in colitis model 3. The probiotics are complementary and alternative medicines and is used for promoting health 4. Some specific probiotic strains, including *Bifidobacterium*, *Saccharomyces*, *Enterococcus*, *Bacillus*, and *Lactobacillus*, have been demonstrated for health benefits 5. Recently, *Lactobacillus* had been reported to ameliorate symptoms of diabetes in human studies 6. *Lactobacillus* also reduced neuropsychiatric disorders, regulated blood pressure, provided physiological benefits, and exerted anti-inflammatory effects 7. The pigmentary disorders and melanoma are abnormal melanogenesis is a feature of many human skin diseases. The wrinkles, melasma and age spots are often observed in skin lighterers in European. In Asia, the care products are used to make skin whiter and moisture 3. Some probiotics is able to serve as a potential care products. Probiotics such as lactobacillus have been demonstrated to modulator skin symptoms 8. *L. plantarum* is effective for alleviating atopic dermatitis symptoms in adults owing to its immunomodulatory effects 9. *L. plantarum* against UVB-induced photoaging in human dermal fibroblasts and hairless mice 10. Meanwhile, *L. plantarum* possesses various pharmacological properties, such as anti-cancer and anti-colitis 11.

We focused on the screening of skin care function from various probiotics for enhancing collagen synthesis and the gene expression of serine palmitoyltransferase small subunit A (SPTSSA). In this study, we sought to investigate that* L. plantarum*-GMNL6 play a role in maintenance skin and wanted to develop *L. plantarum*-GMNL6 as skin care products for the management of skin conditions.

## Materials and Methods

### Cells, bacteria, and reagents

Human skin fibroblast HS68 cells and mouse melanoma B16F10 cells were cultured in Dulbecco's modified Eagle's medium (DMEM) supplemented with 50μg/ml gentamicin, and 10% fetal bovine serum, at 37℃in 5% CO2.* Staphylococcus aureus*,* Propionibacterium acnes*, *Enterococcus faecium*, *L. plantarum*-2, *L. plantarum*-GMNL6, *L. rhamnosus*,* L. paracasei*-2,* L. paracasei*-1, *L. fermentum*-3*, L. fermentum*-2, *L. fermentum*-1, and* L. acidophilus* are available from GenMont Biotech Inc. (Tainan, Taiwan).

### Collagen synthesis assay

HS68 cells were seeded to 6 well plate (2×10^5^ cells/well), washed twice with phosphate buffered saline (PBS), and replaced with serum free medium, and treated with various heat-killing bacteria (5× 10^8^ cells/ml) after 24 h. The supernatant was collected and then analyzed using a Procollagen type I C-peptide EIA kit (TAKARA Bio, Inc, #MK101, Kusatsu , Japan).

### Detection of neural amine synthase gene

HS68 cells were seeded to 6 well plate (2×10^5^ cells/well), and treated with various heat-killing bacteria (5×10^8^ cells/ml) after 24 h. The cell extracts were collected, RNA was extracted and converted into cDNA, and the serine palmitoyltransferase small subunit A (SPTSSA) and the β-actin were quantified by real-time-PCR analysis.

### Purification of Lipoteichoic acid (LTA) and Peptidoglycan (PGN)

*L. plantarum*-GMNL6 was incubated overnight at 37℃. The cells were disrupted using an ultrasonic disruptor (ultrasonic instrument model W-220F) and then centrifuged. After centrifugation, the bacteria were reconstituted with a mixture of chloroform-methanol-water (1:1:0.9), and the aqueous phase containing LTA was collected. The solution was then injected onto Octyl-Sepharose CL-4B column (Sigma) and Q-Sepharose column. The LTA was purified, and stored at -20℃.

The lactic acid bacteria were collected by centrifugation. After washing three times with PBS, 50 g of lactic acid bacteria (wet weight) was broken by an ultrasonic breaker to prepare a crude extract. After centrifugation at 8,000 rpm for 30 minutes, the supernatant was collected and then washed three times with PBS, and centrifuged (8,000 rpm, 30 minutes, 4 ° C), and this procedure was repeated three times. Finally, the precipitates were reacted with 100 mg / ml RNase and 50 mg/ml DNase for 18 h, and then 200 mg/ml trypsin was added, and then reacted at 37℃for 18 h. The precipitates were treated with 5% trichloroacetic acid (TCA). The TCA extract was dried to prepare a peptidoglycan powder.

### Determination of melanin content

The B16F10 cells were harvested and washed twice with PBS. The pelleted cells were lysed in cold lysis buffer (20 mM sodium phosphate pH 6.8, 1% Triton X-100, 1 mM PMSF, and 1 mM EDTA). After centrifugation at 15 000 g for 15 min, the melanin pellets were dissolved in Soluene-350 (Perkin-Elmer, Waltham, MA, USA) for 15 min at 100 ℃. The absorbance at 400 nm was measured 12. The protein content in each sample was determined by bicinchoninic acid (BCA) protein assay (Pierce Biotechnology, Rockford, IL, USA).

### The biofilm formation assay

*S. aureus* was incubated overnight at 37℃. The different concentrations of LTA were treated in to 96 well that had been incubated *S. aureus* (2 ×10^7^ cfu/ well) for 24 hours. The supernatant was removed and washed twice with PBS. The biofilm was fixed with ethanol for 15 min and stained with 0.1% crystal violet. Then, crystal violets were dissolved in acetic acid. The absorbance at 590 nm was measured.

### Study design

A 2-month study was performed. The study was approved by the Ethics Committee of Pingtung Antai Hospital and performed in accordance with the relevant guidelines and regulations. The trial was registered at Clinical Trial.gov with approval NO. NCT03350893. A total of 15 females (Age from 25-50 years old) were recruited. At first visit, participants underwent a baseline examination, including face quality diagnosis, moisturizing test and skin color test. The left face of the participants used base cream and the right face of the participants used base cream including heat-killed *L. plantarum*-GMNL6 (1×10^9^ cells/g cream). The test was used once in the morning and evening for 2 months and every month the face quality (Visia Complexion Analysis, Canfield, USA), moisturizing test and skin color test was measured. Corneometer® CM 825 (Courage+Khazaka electronic GmbH, Köln, Germany) and Skicon 200 EX® (IBS Co., Hamamatsu, Japan) were used determine the hydration level of the skin surface. Derma-Spectrometer (Cortex Technology, Hadsund, Denmark) was used determine the E index (erythema) and M index (melanin) of the skin surface 13. DNA was extracted from the heads of cotton swabs using Quick-DNA Fungal/Bacterial Kit (Zymo research, D6005, CA, USA). Full length 16S rDNA were amplified using forward primers 27F containing the sequence “AGRGTTYGATYMTGGCTCAG” and reverse primers 1492R containing the sequence “RGYTACCTTGTTACGACTT”. At the same time, indexed adapters were added to the ends of the 16S rDNA amplicons using SMRT cell Template Prep Kit (Pacific Biosciences of California, Inc., CA, USA) to generate indexed libraries ready for downstream sequencing on Sequal SMRT cell. DNA libraries were validated by Agilent 2100 Bioanalyzer (Agilent Technologies, Palo Alto, CA, USA), and quantified by Qubit 2.0 Fluorometer. DNA libraries were multiplexed and loaded on PacBio Sequal instrument according to manufacturer's instructions (Pacific Biosciences of California, Inc., CA, USA). PacBio 16S reads were processed using SMRT Analysis.

### Statistical analysis

All data were expressed as mean ± standard deviation (SD). The unpaired, two-tailed Student's t test was used to determine differences between groups. Any P value less than 0.05 is regarded statistically significant.

## Results

### *L. plantarum*-GMNL6 displayed beneficial effects on skin care

**Figure 1:**
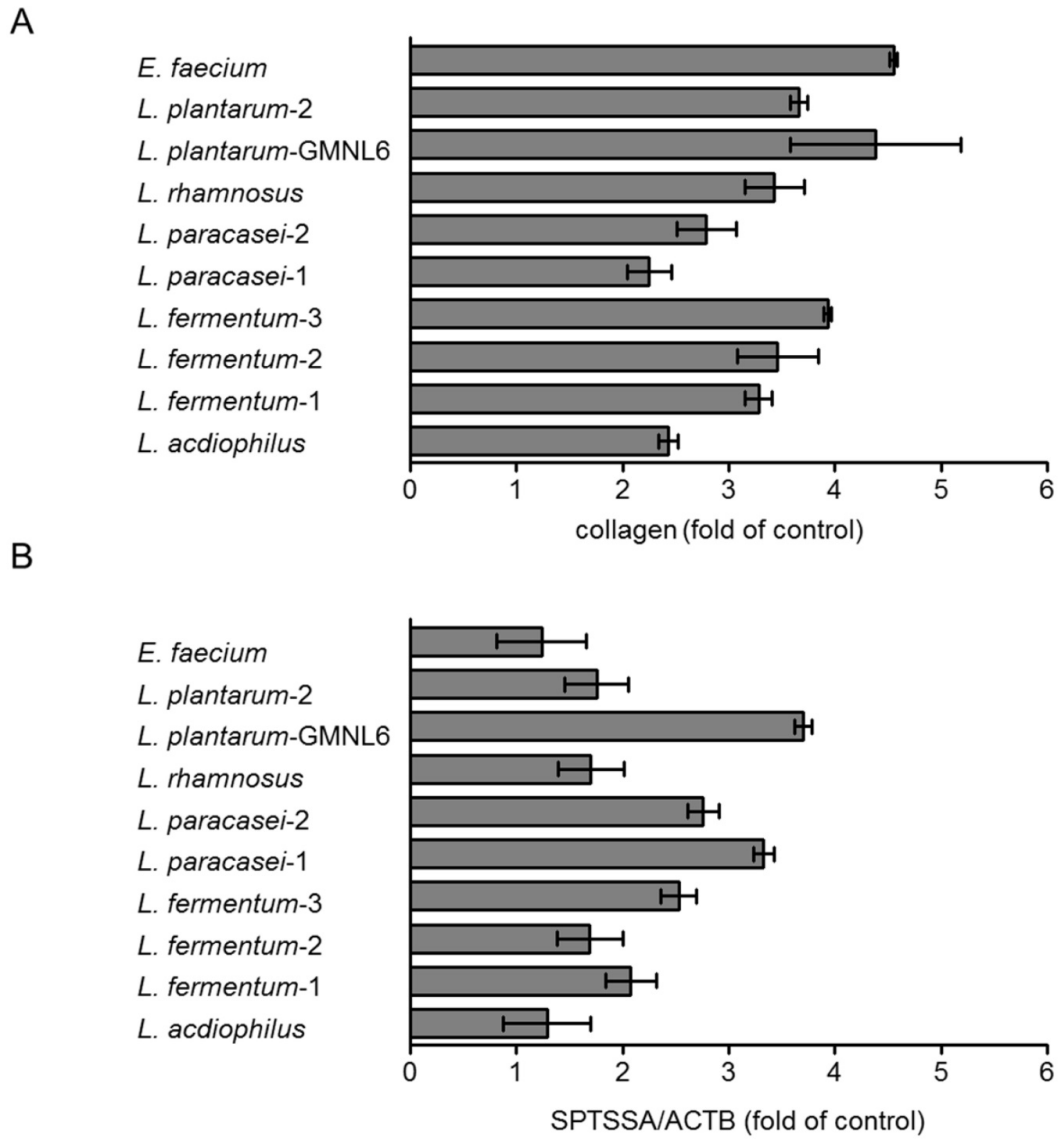
Effects of various probiotic on skin health. HS68 cells were treated with various probiotics (5× 10^8^ cells/ml) after 24 h. (A) The supernatant was collected and then analyzed collagens. (B) The cell extracts were collected, RNA was extracted and converted into cDNA, and the serine palmitoyltransferase small subunit A (SPTSSA) and the β-actin were quantified by real-time-PCR analysis.

In order to find out the optimal probiotics, the various potentially probiotics were treated with HS68 cells by using collagen synthesis and the gene expression of serine palmitoyltransferase small subunit A (SPTSSA) assays. As shown in Figure 1A, the collagen synthesis was significantly increased after* E. faecium* and *L. plantarum*-GMNL6 treatment. The collagen and ceramide are involved in skin rejuvenation. Ceramide is a lipid that exists in the intercellular space of skin cells. The sphingosine and fatty acid constitute ceramide which is the most important and highest proportion of lipids of the skin, with a total content of about 40-50% of the intercellular lipids. Ceramide distributed in the intercellular space and closely maintains the lipid integrity to form a natural waterproof membrane. Ceramide prevents the evaporation of water, allows the keratinocytes to lock in moisture. Serine palmitoyltransferase is the rate-limiting step in ceramide biosynthesis.* L. plantarum*-GMNL6 had the best ability to stimulate the gene expression of SPTSSA compared with other probiotics (Fig. 1B). Dependent on the two assays,* L. plantarum*-GMNL6 was selected in the subsequent analyses.

### The potential components of L. plantarum-GMNL6 involved the beneficial effects on skin care

**Figure 2:**
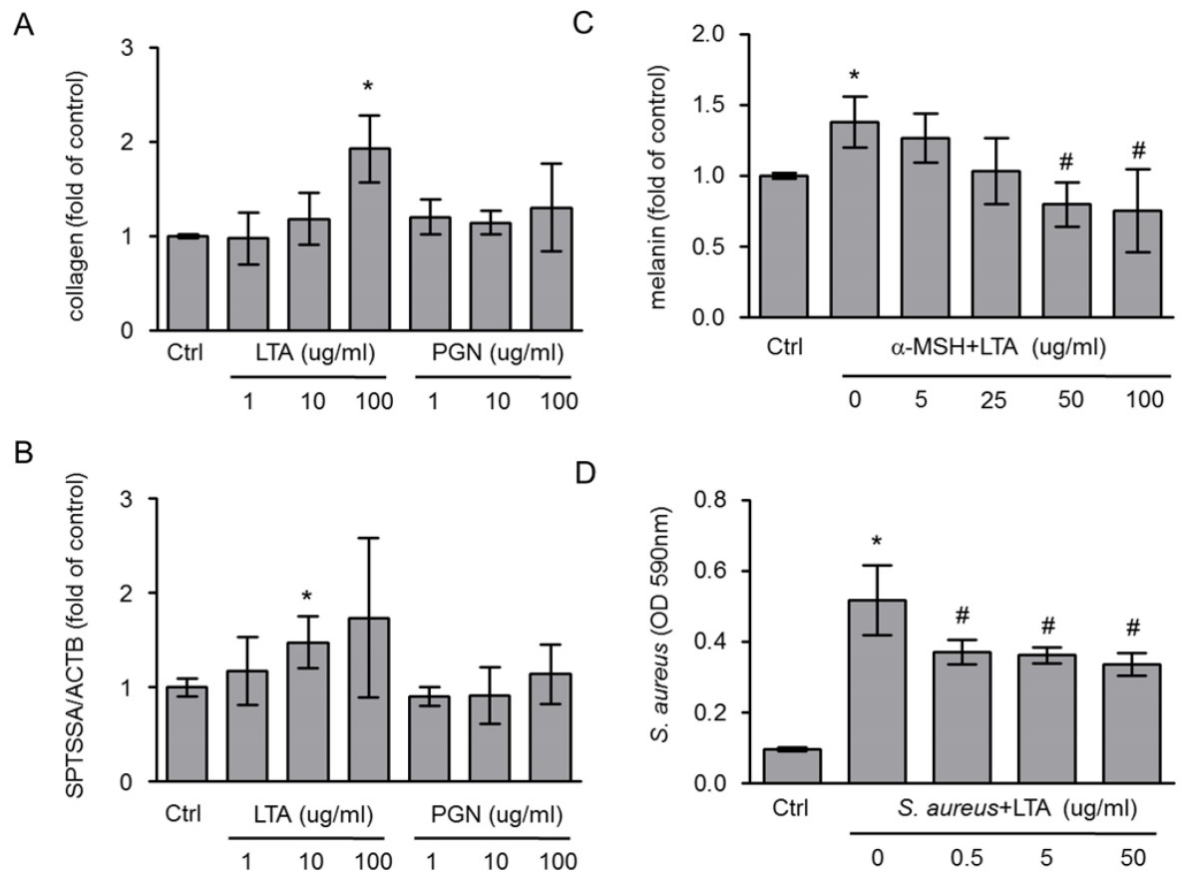
The collagen synthesis, the gene expression of SPTSSA, melanogenesis, and biofilm formation were measured after lipoteichoic acid (LTA) and peptidoglycan (PGN) of *L. plantarum*-GMNL6 treatment. Effect of LTA and PGN on (A) cellular collage synthesis and (B) the gene expression of SPTSSA. (C) Effect of lipoteichoic acid (LTA) and peptidoglycan (PGN) on cellular melanin content. (D)The inhibition of biofilm formation of *S. aureus* was measured after LTA and PGN treatment. (n = 6, data are mean± SD. * P < 0.05, ^#^ P < 0.01).

Because we used the death bacteria to treat the cells, we proposed that the cell wall components played an important role in collagen synthesis and SPTSSA gene expression. The lipoteichonic acid (LTA) and peptidoglycan (PGN) are the major cell wall components of *Lactobacillus* that had be demonstrated the pivotal components for beneficial effects on host 14. First, the collagen synthesis and SPTSSA gene expression were used to evaluate the function of cell wall components (LTA and PGN). Although PGN slightly increased collagen synthesis and SPTSSA gene expression in HS68 cells, the LTA of *L. plantarum*-GMNL6 had significant effects on collagen synthesis and SPTSSA gene expression (Fig 2 A and B). Herein, the results suggest that LTA of* L. plantarum*-GMNL6 involved in skin health. Meanwhile, mouse B16F10 melanoma cells were used to investigate the anti-melanogenic activity of the LTA of *L. plantarum*-GMNL6. The melanin content after B16F10 cells treated with various concentrations LTA are shown in Fig 2 C. *Staphylococcus aureus* is commonly found on the surface of skin. The LTA of *L. plantarum-*GMNL6 significantly inhibited the biofilm formation of *S. aureus* in a dose-dependent manner (Fig. 2D).

### A heat-killed L. plantarum-GMNL6 in clinical trail

**Figure 3:**
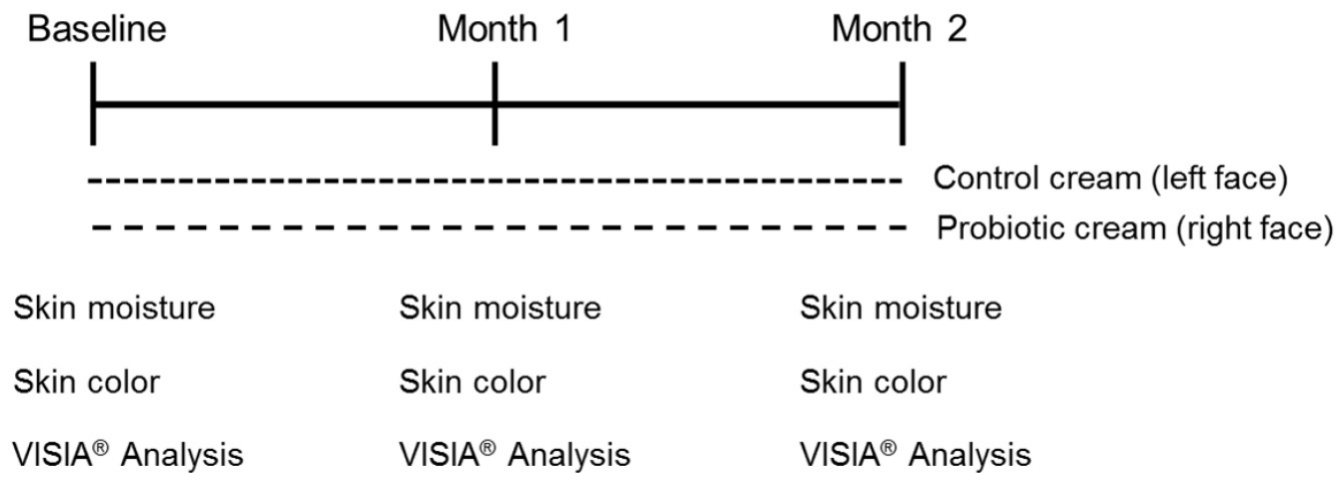
Recruitment of participants and the study design. The cream containing probiotics or not was performed a total of 2 months. All participates were requested to collect data by using Corneometer^®^ CM 825, Skicon 200 EX® Derma-Spectrometer, and VISIA^®^.

The study design is shown in Fig. 3. A total of 15 females (Age from 25-50 years old) were recruited. At first visit, participants underwent a baseline examination, including face quality diagnosis, moisturizing test and skin color test. The left face of the participants used base cream and the right face of the participants used base cream including heat-killed* L. plantarum*-GMNL6 (1×10^9^ cells/g cream). We hypothesized that heat-killed* L. plantarum*-GMNL6 could interact with skin microbiota and acquire different microbial communities that might finally result in successful skin health. To examine the relationship between the skin microbiota and heat-killed *L. plantarum-*GMNL6 treatment, we performed 16S rDNA-based sequencing to analyze the bacterial abundance and composition in the human skin. *Propionibacterium* was significantly lowered in the* L. plantarum*-GMNL6 treatment. *Streptococcus* and *Staphylococcus* also showed higher abundance compared with no treatment. Furthermore, the test was used once in the morning and evening for 2 months and every month the face quality, moisturizing test and skin color test was measured. Corneometer® CM 825 and Skicon 200 EX® were used determine the hydration level of the skin surface. Derma-Spectrometer was used determine the E index (erythema) and M index (melanin) of the skin surface. As shown in Table 1, the hydration levels of the heat-killed *L. plantarum*-GMNL6 treated skin surface were significantly higher than these of control group. The hydration levels of the skin surface were similar by using different instruments. Meanwhile, the heat-killed *L. plantarum*-GMNL6 had the ability to reduce the erythema and melanin (Table 1). Furthermore, by using Visia to detect the face quality, the wrinkles, texture, and UV spots had been significantly improved after heat-killed *L. plantarum*-GMNL6 treatment (Table 2). The porphyrins of treated face were reduced compared with non-treated face. Taken together, the cream that added heat-killed *L. plantarum*-GMNL6 can improve the face quality.

## Discussion

Probiotics are considered important roles in skin health, wellbeing, and microbiome-associated skin disease. Therefore, we explored the possibility that probiotics associated with skin health. Supplementation of the *L. plantarum*-GMNL6 to cell improved the collagen synthesis and moisturizing. Although probiotic cell wall components have been demonstrated to be related to probiotic function 15, very few evidences proposes that they contribute for skin care. In the present study, the LTA of *L. plantarum*-GMNL6 was involved in improvement of skin condition, including collagen synthesis, moisturizing, melanogenesis, and anti-biofilm formation. In human studies, some people have been facing a lot of ordeals at skin condition. After two months *L. plantarum*-GMNL6 treatment, we observed that some skin conditions had been improved in human.

Unlike LTA from other Gram-positive bacteria, LTA from *L. plantarum*-GMNL6 acts as cosmetic regulator substance. In accordance with previous observations, the LTA of* Lactobacillus* has multi-functions, including anti-inflammatory, improvement of diarrhea, and blood glucose control 7. Previously, we found that an extract of *R. sphaeroide*s inhibited proinflammatory cytokines and enhanced mucosal immune balance in a dextran sodium sulfate (DSS)-induced colitis model 3, 16. We also demonstrated the extract of *R. sphaeroides* as an anti-melanogenic agent with the capacity to ameliorate α-MSH induced-hyperpigmentation 17.

The bacteria produce compounds that are widely used in cosmetic and pharmaceutical applications. Microbe-based therapies cosmetic has been noticed. The mitogen-activated protein kinase (MAPKs) signaling pathway involved in the melanogenesis. The activation of protein kinase B (AKT) is responsible for suppression for melanin synthesis 18. The LTA of *L. plantarum*-GMNL6 may affect the signaling to reduce the melanogenesis. The people *L. plantarum*-GMNL6 had better skin conditions after 1 month L. plantarum-GMNL6 treatment (Fig. S1). Further work is warranted to elucidate the molecular mechanism of L. plantarum-GMNL6 for hypopigmentation. These findings point out that LTA of L. plantarum-GMNL6 might contribute to its therapeutic effect on anti-melanogenesis.

## Supplementary Material

Supplementary figure.

## Figures and Tables

**Figure 4:**
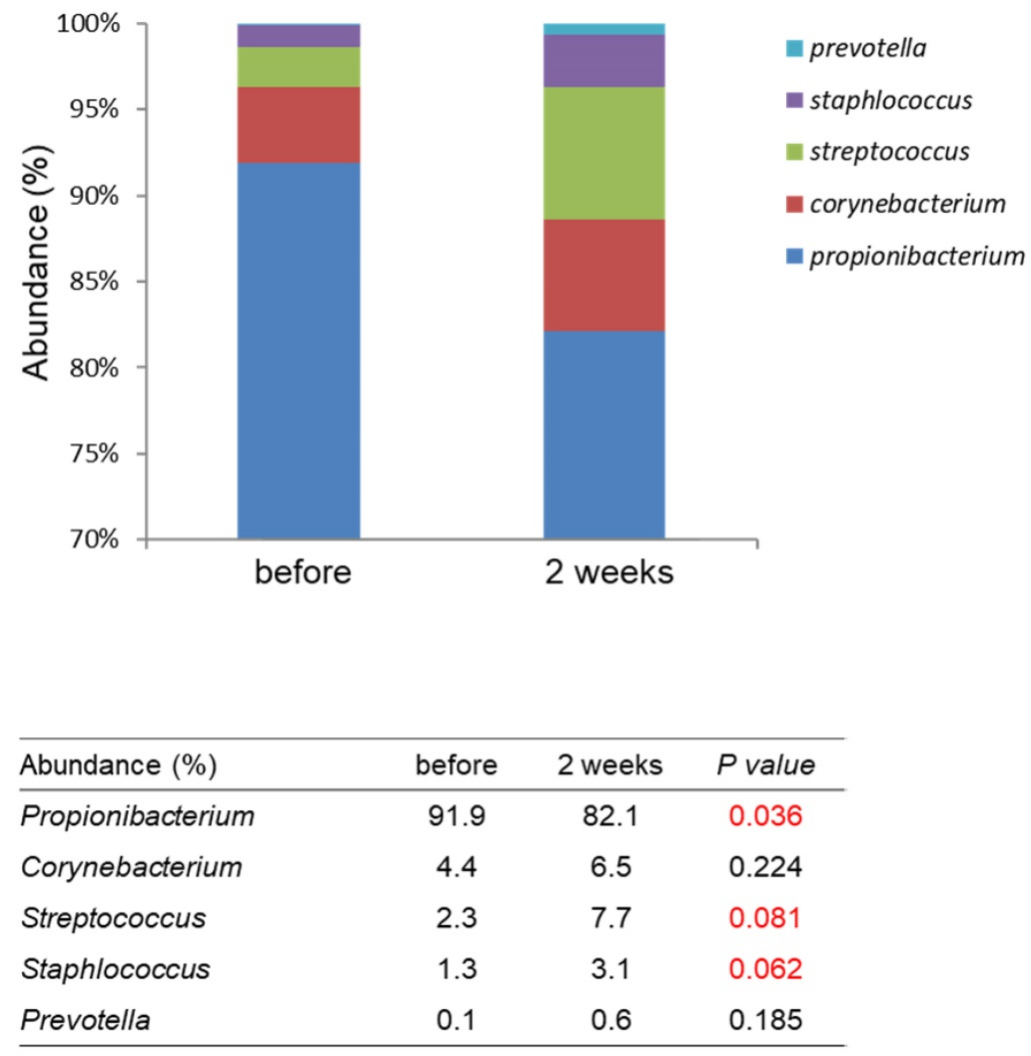
Comparison with the skin microbiomes before and after using *L. plantarum*-GMNL6 treatment. The relative abundance of five major genera in skin microbiomes is compared before and after using *L. plantarum*-GMNL6 treatment. Statistically significant P-values are shown in red.

**Table 1:**
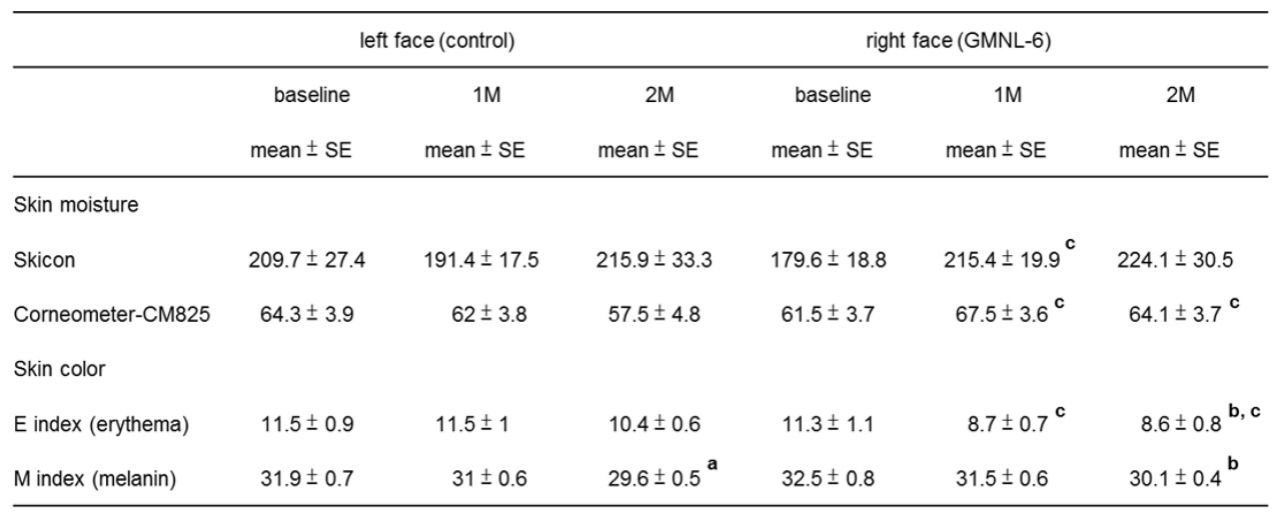
The effects of *Lactobacillus plantarum*-GMNL6 on human skin health.

a: comparing with baseline in left face; P < 0.05 was considered statistically significant. b: comparing with baseline in right face; P < 0.05 was considered statistically significant. c: comparing with left face at the same time period; P < 0.05 was considered statistically significant.

**Table 2:**
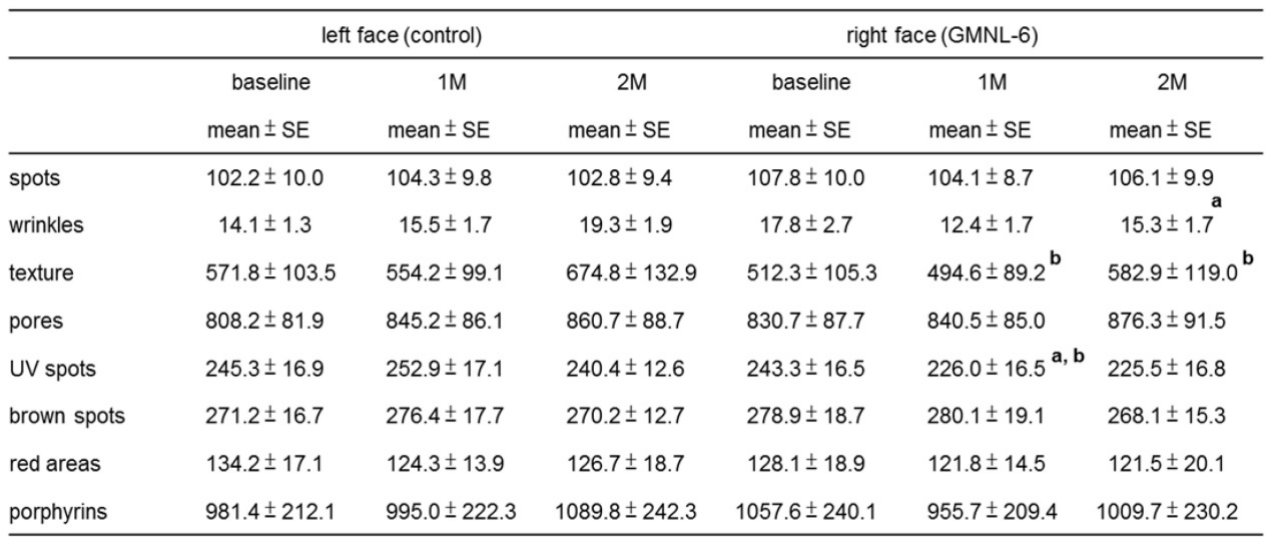
The effects of *Lactobacillus plantarum*-GMNL6 on human skin health.

a: comparing with baseline in right face; P < 0.05 was considered statistically significant.

## References

[1] Huang WC, Wei CC, Huang CC, Chen WL, Huang HY (2019). The beneficial effects of Lactobacillus plantarum PS128 on high-intensity, exercise-induced oxidative stress, inflammation, and performance in triathletes. Nutrients.

[2] Okamoto K, Fujiya M, Nata T (2012). Competence and sporulation factor derived from Bacillus subtilis improves epithelial cell injury in intestinal inflammation via immunomodulation and cytoprotection. Int J Colorectal Dis.

[3] Liu WS, Chen MC, Chiu KH, Wen ZH, Lee CH (2012). Amelioration of dextran sodium sulfate-induced colitis in mice by Rhodobacter sphaeroides extract. Molecules.

[4] Qian L, Gao R, Huang J, Qin H (2019). Supplementation of triple viable probiotics combined with dietary intervention is associated with gut microbial improvement in humans on a high-fat diet. Exp Ther Med.

[5] Tsai YL, Lin TL, Chang CJ, Wu TR, Lai WF, Lu CC, Lai HC (2019). Probiotics, prebiotics and amelioration of diseases. J Biomed Sci.

[6] Hsieh MC, Tsai WH, Jheng YP, Su SL, Wang SY, Lin CC, Chen YH, Chang WW (2018). The beneficial effects of Lactobacillus reuteri ADR-1 or ADR-3 consumption on type 2 diabetes mellitus: a randomized, double-blinded, placebo-controlled trial. Sci Rep.

[7] Chang CJ, Lin TL, Tsai YL, Wu TR, Lai WF, Lu CC, Lai HC (2019). Next generation probiotics in disease amelioration. J Food Drug Anal.

[8] Muizzuddin N, Maher W, Sullivan M, Schnittger S, Mammone T (2012). Physiologic effect of a probiotic on the skin. J Cosmet Sci.

[9] Prakoeswa CRS, Bonita L, Karim A, Herwanto N, Umborowati MA, Setyaningrum T, Hidayati AN, Surono IS (2020). Beneficial effect of Lactobacillus plantarum IS-10506 supplementation in adults with atopic dermatitis: a randomized controlled trial. J Dermatolog Treat.

[10] Kim HM, Lee DE, Park SD, Kim YT, Kim YJ, Jeong JW, Jang SS, Ahn YT, Sim JH, Huh CS, Chung DK, Lee JH (2014). Oral administration of Lactobacillus plantarum HY7714 protects hairless mouse against ultraviolet B-induced photoaging. J Microbiol Biotechnol.

[11] Moon PD, Lee JS, Kim HY, Han NR, Kang I, Kim HM, Jeong HJ (2019). Heat-treated Lactobacillus plantarum increases the immune responses through activation of natural killer cells and macrophages on in vivo and in vitro models. J Med Microbiol.

[12] Phacharapiyangkul N, Thirapanmethee K, Sa-Ngiamsuntorn K, Panich U, Lee CH, Chomnawang MT (2019). Effect of Sucrier Banana Peel extracts on Inhibition of melanogenesis through the ERK signaling pathway. Int J Med Sci.

[13] Clarys P, Clijsen R, Barel AO (2011). Influence of probe application pressure on in vitro and in vivo capacitance (Corneometer CM 825(®)) and conductance (Skicon 200 EX(®)) measurements. Skin Res Technol.

[14] Lee IC, Tomita S, Kleerebezem M, Bron PA (2013). The quest for probiotic effector molecules-unraveling strain specificity at the molecular level. Pharmacol Res.

[15] Kim KW, Kang SS, Woo SJ (2017). Lipoteichoic acid of probiotic Lactobacillus plantarum attenuates Poly I:C-induced IL-8 production in porcine intestinal epithelial cells. Front Microbiol.

[16] Chang WW, Liu JJ, Liu CF, Liu WS, Lim YP, Cheng YJ, Lee CH (2013). An extract of Rhodobacter sphaeroides reduces cisplatin-induced nephrotoxicity in mice. Toxins (Basel).

[17] Liu WS, Kuan YD, Chiu KH, Wang WK, Chang FH, Liu CH, Lee CH (2013). The extract of Rhodobacter sphaeroides inhibits melanogenesis through the MEK/ERK signaling pathway. Mar Drugs.

[18] Tsao YT, Huang YF, Kuo CY (2016). Hinokitiol inhibits melanogenesis via AKT/mTOR signaling in B16F10 mouse melanoma cells. Int J Mol Sci.

