# Supplementary figures and images for: Regulatory effects of Lactobacillus plantarum-GMNL6 on human skin health by improving skin microbiome
Source: Int J Med Sci. 2021 Jan 1;18(5):1114–20. doi: 10.7150/ijms.51545 (PMC7847631; doi:10.7150/ijms.51545)

Figure supplement 1

【0M】

【1M\_GMNL-6 cream】

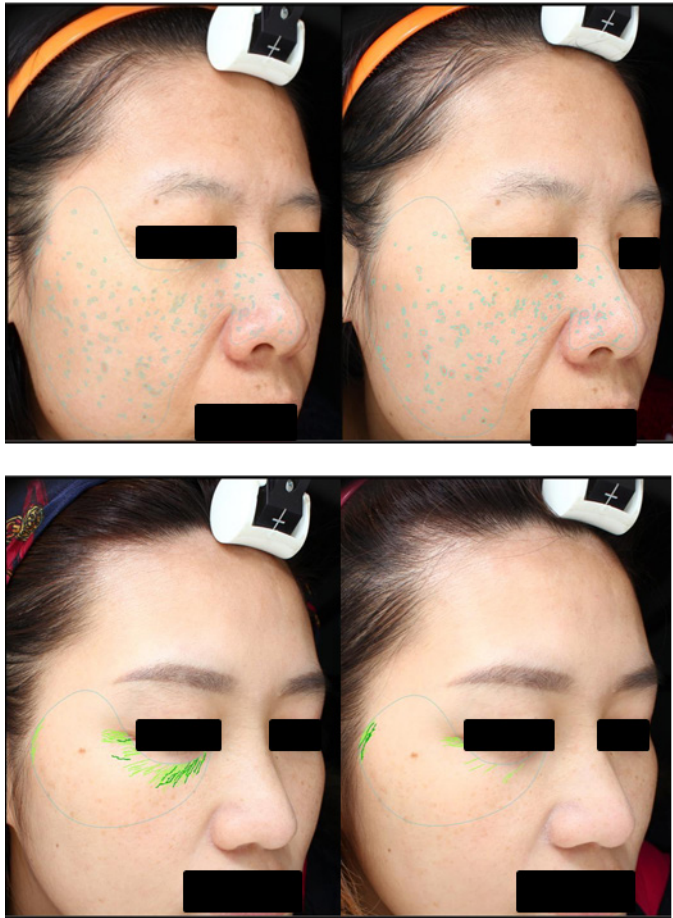

Supplement: Supplementary file 1 — Supplementary figure. [file ijmsv18p1114s1.pdf]
